# Anatomy Based Networks and Topology Alteration in Seizure-Related Cognitive Outcomes

**DOI:** 10.3389/fnana.2018.00025

**Published:** 2018-04-06

**Authors:** Qian Wu, Charlie W. Zhao, Zhe Long, Bo Xiao, Li Feng

**Affiliations:** ^1^Department of Neurology, First Affiliated Hospital, Kunming Medical University, Kunming, China; ^2^Department of Neuroscience, Yale School of Medicine, Yale University, New Haven, CT, United States; ^3^Sydney Medical School and the Brain & Mind Institute, The University of Sydney, Camperdown, NSW, Australia; ^4^Department of Neurology, Xiangya Hospital, Central South University, Changsha, China

**Keywords:** epilepsy, cognition, networks, topology, seizure

## Abstract

Epilepsy is a paroxysmal neurological disorder characterized by recurrent and unprovoked seizures affecting approximately 50 million people worldwide. Cognitive dysfunction induced by seizures is a severe comorbidity of epilepsy and epilepsy syndromes and reduces patients’ quality of life. Seizures, along with accompanying histopathological and pathophysiological changes, are associated with cognitive comorbidities. Advances in imaging technology and computing allow anatomical and topological changes in neural networks to be visualized. Anatomical components including the hippocampus, amygdala, cortex, corpus callosum (CC), cerebellum and white matter (WM) are the fundamental components of seizure- and cognition-related topological networks. Damage to these structures and their substructures results in worsening of epilepsy symptoms and cognitive dysfunction. In this review article, we survey structural, network changes and topological alteration in different regions of the brain and in different epilepsy and epileptic syndromes, and discuss what these changes may mean for cognitive outcomes related to these disease states.

## Introduction

Epilepsy is a prevalent neurological disorder characterized by recurrent seizures. An important complication of epilepsy and epilepsy syndromes is disruption in cognitive ability. Understanding how cognitive dysfunction occurs will not only improve the quality of life for patients, but will also help elucidate neural mechanisms underlying cognition.

There are many forms of epilepsy, broadly characterized as involving partial or generalized seizures. Epilepsy syndromes are defined by associated clinical manifestations. Childhood absence epilepsy (CAE), for instance, is characterized by brief, consciousness-impairing staring spells and 3-Hz discharges on EEG.

Some forms of epilepsy and epilepsy syndromes may lead to cognitive impairment, further compounding disease burden. Yet, how cognitive impairment relates to seizures is unclear. How do seizures impact cognition? What are the neural mechanisms mediating this relationship? These are important questions being addressed with investigation on multiple levels.

In this review article, we focus on changes to neuroanatomy and neural networks that alter the topological relationship between brain regions. These alterations result in greater seizure susceptibility and cognitive dysfunction. Furthermore, increased seizure severity causes further topological changes, thus establishing a vicious cycle. In this survey, we focus on specific brain areas involved in seizure initiation and cognition and epilepsy syndromes associated with cognitive impairment. Abbreviations used throughout this review article are in Table 1. A summary of structural and functional anatomic findings associated with major epilepsy syndromes covered in this review article can be found in Table 2.

**Table 1:** A list of abbreviations used in this review article.

Abbreviation	Meaning
AHC	Amygdalo-hippocampal complex
BECTS	Benign epilepsy with centro-temporal spikes
CA	Cornu ammonis (region of the hippocampus)
CAE	Childhood absence epilepsy
CC	corpus callosum
CPS	Complex partial seizure
DMN	Default mode network
DTI	Diffuse tensor imaging
ELS	Early life seizures
FDG-PET	Fluorodeoxyglucose positron emission tomography
FLE	Frontal lobe epilepsy
FS	Febrile seizure
HS	Hippocampal sclerosis
JME	Juvenile myoclonus epilepsy
LSTGp	Left posterior superior temporal gyrus
mTLE	Medial temporal lobe epilepsy
PFC	Prefrontal cortex
rTMS	Repetitive transcranial magnetic stimulation
SMA	Supplemental motor area
STP	Short term potentiation
TLE	Temporal lobe epilepsy
WM	White matter

**Table 2:** Structural and functional anatomic changes involved in the epilepsy syndromes covered in this review article.

Condition	Structural	Functional
Early life seizures	*Neuronal injury in l. septal nuclei, amygdala, v. subiculum/CA1*	*CA1 unable to form spatial mapPrefrontal cortex short term potentiation alterations (LII/LIII-to-LV and LV-to-LV)*
Temporal lobe epilepsy	Volume loss in thalamus, hippocampus, cerebellum Temporal and frontal cortical thinning (only frontal in some studies) With postictal psychosis: prefrontal and temporal thickening	Lateral amygdala stimulation causes experiential symptoms White matter abnormalities in *a*. and *m*. temporal, *ips*. cerebellum, *p*. callosum, *con*. frontoparietal Interictal hypometabolism in epileptic region Poorly segregated cognitive modules
Temporal lobe epilepsy with hippocampal sclerosis	*Neuronal loss and gliosis centered around CA1 Granule cell dispersion and loss and sprouting of interneurons in dentate gyrus* Mesiotemporal cortex sclerosis, neuronal loss, gliosis Increase in complexity of temporal and frontal cortical folding Cortical thinning in regions connected with hippocampus	*Decrease connectivity between amygdalo-hippocampal complex and bil. v. prefrontal, temporal pole, con. p. cingulate; Increase connectivity with a. cingulate, d. m. prefrontal, bil. temporo-parietal junction* Increased synchronization in ips. parahippocampus, midbrain, insula, callosum, bil. sensorimotor cortex, frontoparietal subcortical structures; Decreased synchronization in cerebellum, precuneus, p. cingulate, bil. i. l. parietal, m. PFC
Frontal lobe epilepsy	Frontal cortical volume loss in children for left FLE: (ips) s. frontal, paracentral, precuneus, cingulate, i. parietal, supramarginal, postcentral, s. temporal (con) s. and m. frontal, m. orbitofrontal, supramarginal, postcentral, s. temporal banks, parahippocampus; right FLE: (ips) precentral, postcentral, transverse temporal, parahippocampus, lingual, l. occipital (con) s. front, i. parietal, postcentral, s. temporal, p. cingulate, lingual	Frontal, temporal, parietal hypometabolism
Juvenile myoclonic epilepsy	Gray matter changes in s. m. frontal, p. cingulate and a. callosum Gray matter reduction in supplementary motor area and p. cingulate	White matter abnormalities in bil. a. and s. corona radiata, callosum genu and body, cingulum-temporal connections, p. parietal, and frontal Reduced connectivity between prefrontal and frontopolar regions; Increased connectivity between occipital cortex and supplementary motor area Hyperconnectivity in subnetwork involving primary motor cortex, precuneus, cerebellum lobules IV and V, basal ganglia, bil. parietal/postcentral, subcortical regions and right hippocampus
Childhood absence epilepsy		White matter abnormalities in bil. thalamus, a. callosum, upper brainstem, prefrontal, a. cingulate, parietal, p. cerebellum, bil. putamen, bil. p. internal capsule Altered whole-brain topology Impaired subcortical and orbitofrontal subnetworks Microstructural changes in callosum and bil. precuneus

*Some changes span both categories, in which case we classified it according to its dominant feature. Abbreviations used are positional: a-anterior, p-posterior, v-ventral, d-dorsal, s-superior, i-inferior, l-lateral, m-medial, ips-ipsilateral, con-contralateral, bil-bilateral. Italicized text are animal studies*.

## Anatomical Basis of Seizure-Related Cognitive Outcomes

### Hippocampal Dysfunction

Animal and clinical studies show that seizures can result in hippocampal dysfunction associated with cognitive and behavioral disturbances in both the developing and mature brain (Alvarez et al., 2014). The hippocampus plays an important role in memory formation. Signals from the occipital, temporal and parietal lobes, posterior cingulated cortex and the contralateral hippocampus converge on hippocampal neurons through the medial and lateral perforant pathways and through the anterior commissure (Aggleton, 2012). Additional synaptic integration arises from local recurrent excitatory networks, where feedforward and inhibitory connections contribute to the acquisition of new episodic memories (Casanova et al., 2014).

Seizures have been shown to cause aberrant neurogenesis in the hippocampus and form faulty circuits that disrupt hippocampal function (Fournier et al., 2013). Aberrant neurogenesis, including neural progenitor proliferation, ectopic granule cell production and mossy fiber sprouting after seizures, are harmful to new memory formation (Arnold et al., 2016). In a genetic mouse model, blocking ectopic granule cell production using Nestin-δ-HSV-thymidine kinase-EGFP before acute seizures reduces the frequency of spontaneous recurrent seizures and improves epilepsy-associated memory deficit (Cho et al., 2015). In addition, brief recurrent seizures induced by flurothyl and the tetanus toxin result in dramatic impairment of hippocampus-based spatial learning and memory (Nishimura et al., 2011). Place cells in cornu ammonis 1 (CA1) are unable to form a stable spatial map when animals experience early life seizures (ELS; Karnam et al., 2009). In the long-term kindling rat model, seizures during cell maturation prevent newborn neurons from integrating properly into hippocampal circuits that are important for memory formation. Reducing activation of adult-born neurons may be a therapeutic strategy to reverse cognitive deficits in epileptic syndromes (Fournier et al., 2013).

In temporal lobe epilepsy (TLE), extra-hippocampal volume abnormalities are observed in association with cognitive dysfunction. Chronic TLE is characterized by extra-hippocampal brain abnormality and cognitive impairment in both memory and non-memory domains (Tuchscherer et al., 2010). Baseline thalamic volumes of TLE patients are lower than that of controls and executive functioning is poorer in the TLE group even after correcting for hippocampal and frontal lobe volumes (Tuchscherer et al., 2010). The thalamus is involved in the evolution and propagation of partial seizures and plays a role in cognition. The anterior thalamus interacts with the hippocampus and cortex and functions in memory processing and spatial navigation, the intralaminar thalamic nucleus and the parafascicular thalamus are involved in behavioral flexibility, and the mediodorsal thalamic nucleus in goal-directed behavior (Saniya et al., 2017). The amygdala also affects cognitive tasks such as emotional appraisal. The lateral amygdala, which when stimulated causes experiential symptoms in TLE patients, projects to the hippocampus and temporal neocortex, and selective amygdalotomy can be used to effectively treat TLE (Saniya et al., 2017).

Plasticity and metaplasticity are thought to be fundamental to learning and memory and may be involved in epilepsy. Synaptic plasticity is modulated by prior synaptic activity, a phenomenon termed metaplasticity (Zhang and Luo, 2011). Dysfunction of plasticity and metaplasticity in the hippocampus is implicated in febrile seizure (FS), a common childhood episode that can impair cognitive function (Zhang and Luo, 2011). FS impairs metaplasticity of the lateral perforant path of the rat hippocampus without affecting long-term potentiation, suggesting that FS may cause dysfunction in the excitatory status of other pathways that ultimately lead to brain damage (Zhang and Luo, 2011).

In the following sections, we further explore the relationship between seizures and hippocampal dysfunction, specifically focusing on Hippocampal sclerosis (HS), dendritic pathology and cortical alterations.

#### Hippocampal Sclerosis

HS is a pathological development associated with benign and drug-resistant mTLE and is involved in epilepsy-related cognitive dysfunction (Blumcke et al., 2012). Unilateral HS is the most frequent mTLE pathological change, observed in 60%–80% of mTLE patients (Berkovic et al., 1995). HS is also observed in patients who suffer from neurodegenerative diseases including Alzheimer’s disease and fronto-temporal lobe dementia (Bandopadhyay et al., 2014).

Histologically, epileptic HS exhibits a consistent pattern of neuronal loss and gliosis centered on the CA1 subfield and a more variable loss in CA4 and other subfields (Thom, 2009). Other features seen in epileptic HS include the dispersion of the granule cell layer of the dentate gyrus and sprouting of mossy fiber axons (Schmeiser et al., 2017). Loss and sprouting of dentate gyrus interneurons in either a unilateral or bilateral pattern are also observed in post mortem (Martinian et al., 2012; Thom et al., 2012). Interneuronal axon sprouting is a functional process related to network changes through synaptic re-organization (Maglóczky, 2010).

In unilateral mTLE with HS, seizures can induce effective connectivity alterations between the non-epileptic amygdalo-hippocampal complex (AHC) and the rest of human brain (Trotta et al., 2013). These changes include a significant decrease in connectivity between the non-epileptic AHC and the bilateral ventral prefrontal cortical areas, the temporal pole and the posterior cingulated cortex contralateral to HS, and a significant increase in connectivity between the non-epileptic AHC and midline structures such as the anterior cingulate and dorsal medial prefrontal cortices, as well as the bilateral temporo-parietal junction (Trotta et al., 2013). Connectivity alterations also exist between the non-epileptic AHC and some limbic and default mode network (DMN) areas. These changes may account for the emotional, cognitive and decision-making impairment that occur frequently in mTLE patients (Trotta et al., 2013).

#### Dendritic Pathology

Abnormalities in dendritic spines are often observed in brain specimens from epilepsy patients and animal models of epilepsy (Bartsch et al., 2010). Dendritic spine density progressively decreases with increasing duration of infancy- or childhood-onset epilepsy (Casanova et al., 2014). However, the relationship between dendritic pathology and epileptogenesis and cognitive deficits is complex (Wong and Guo, 2013).

Dendritic spines are small actin rich dendritic protrusions that receive excitatory input from axons and presynaptic signals. They are the major sites of contact for synapsing neurons and provide a metric for the number and strength of signaling connections between the elements of a functional neuronal circuit (Mancuso et al., 2014). Epilepsy or seizures damage these structures, thus contributing to progressive epileptogenesis, decreased seizure thresholds and learning and memory problems (Wong and Guo, 2013). In turn, dendritic spine abnormalities can promote hyperexcitability in circuits, further worsening the epilepsy and associated cognitive dysfunction. Using a morphologically and biophysically realistic model of a bursting layer 5 pyramidal cell from the cat visual cortex, van Elburg and van Ooyen (2010) showed that alterations in size or topology of pyramidal cell morphology, such as shortening or lengthening the dendritic tree, or even just modifying the pattern in which the branches in the tree are connected, may change neuronal burst firing and affect information processing and cognition. This resembles the neuronal mechanism seen in Alzheimer’s disease, mental retardation, epilepsy and chronic stress (van Elburg and van Ooyen, 2010).

Recurrent ELS may suppress dendrite growth by impairing the addition of new branches and/or impairing the growth of existing branches (Casanova et al., 2012). Apical and basilar dendrites appear to respond differently to seizures. Recurrent ELS do not affect the length or branch number of apical dendrites but reduce that of basilar dendrites in mouse models (Nishimura et al., 2011; Yang et al., 2015). The latter may reduce anatomical and molecular substrates for learning and memory. Even though apical dendrite morphology appear unaffected, seizures may still produce activity-dependent alterations in synaptic efficacy and affect aspects of hippocampal synaptic plasticity, such as long-term potentiation and depression, which are thought to form the cellular basis for hippocampal learning and memory (Alarcon et al., 2006).

Interestingly, the effect of seizures on dendrite anatomy and spatial learning appears to be age-dependent. The effects mentioned above are absent when seizures are induced in older rats. Thus, there may exist a neurodevelopmental time window during which dendritic growth and maintenance are particularly vulnerable to seizures. This would explain the cognitive impairment seen in children with epilepsy and in adult patients who have had early-onset epilepsy (Hermann et al., 2008).

### Cortical Changes

We next discuss cortical changes in TLE, frontal lobe epilepsy (FLE), ELS and juvenile myoclonus epilepsy (JME). In TLE, cortical parameters including area, thickness and volume are affected, and cognitive impairment in TLE is associated with the severity of cortical abnormalities (Gutierrez-Galve et al., 2012). Many studies report cortical abnormalities in the temporal and frontal regions (Raj et al., 2010; Voets et al., 2011) while some found abnormalities only in the frontal lobe (Gutierrez-Galve et al., 2012). This discrepancy may arise from differences in inclusion criteria and methodology of the studies, thus more evidence is needed for clarification. In general, in TLE with or without HS, cortical thinning is associated with loss of volume in the hippocampus and anterior thalamus (Mueller et al., 2010).

TLE patients with HS is associated with neuropathology primarily in the frontotemporal cortex, which includes mesiotemporal sclerosis, neuronal loss and gliosis (Kaaden et al., 2011). These extratemporal cortical abnormalities contribute to patterns of cognitive impairment in TLE, including IQ decline and deficits in language, executive and motor functions (Hermann et al., 2009; Keller et al., 2009; Tuchscherer et al., 2010). Surface-based morphometry reveals an increase in the complexity of temporal and frontal cortical folding distant from the epileptic focus (Voets et al., 2011) and widespread cortical thinning in regions connected with the hippocampus (Raj et al., 2010).

In TLE with postictal psychosis, cortical thickness is increased in prefrontal and temporal regions (DuBois et al., 2011). Cortical abnormalities in extratemporal areas in TLE patients may not only explain the observed cognitive impairment, but also reflect the spread of seizure activity through the thalamus in addition to limbic pathways (Bernhardt et al., 2008).

FLE and its corresponding cognitive outcomes are associated with changes in cortical parameters. A study using ^18^F-Fluorodeoxyglucose positron emission tomography (FDG-PET) in children with FLE shows widespread hypometabolism not only in the frontal lobe but also in the extrafrontal cortex, including the temporal and parietal cortices (da Silva et al., 1997). This suggests that, similar to TLE, FLE seizures also spread from a single epileptic focus to brain regions via pathways including cortico-cortical connections.

Frontal cortical volumes are smaller in children with intractable FLE than in healthy children (Lawson et al., 2002) with similar changes seen in extrafrontal areas (Widjaja et al., 2011), which may explain the complicated cognitive dysfunction in intractable FLE. Surface-based morphometry shows that in children with left FLE, cortical thinning is present in the left superior frontal, paracentral, precuneus, cingulate, inferior parietal, supramarginal, postcentral and superior temporal gyri, as well as in the right superior and middle frontal, medial orbitofrontal, supramarginal, postcentral, banks of superior temporal sulcus and parahippocampal gyri. Conversely, in children with right FLE, cortical thinning is present in the right precentral, postcentral, transverse temporal, parahippocampal, lingual and lateral occipital gyri, as well as in the left superior frontal, inferior parietal, postcentral, superior temporal, posterior cingulate and lingual gyri (Widjaja et al., 2011). Widespread cortical thinning and gray matter volume reduction appear to be more prominent in left FLE than in right FLE (Widjaja et al., 2011). We note that although cortical abnormalities are observed in FLE, there is currently no direct evidence linking these changes and cognitive dysfunction in FLE patients.

The prefrontal cortex (PFC) is an important brain region for neonatal seizure-related cognitive impairment, as in the case of ELS. Anatomical and electrophysiological studies show that direct connections between the hippocampus and medial PFC as part of the hippocampo-PFC circuit play an important role in aspects of learning and memory processing including information consolidation and working memory (Preston and Eichenbaum, 2013). Even a single episode of neonatal seizure can permanently alter synaptic organization and transmission (Isaeva et al., 2006) in the CA1 region of the hippocampus and impair spatial learning and working memory (Kleen et al., 2011b). Animals that experience ELS display a deficit in behavioral flexibility associated with PFC architectural changes (Kleen et al., 2011a). ELS may also result in anxiety-like behavior and impaired spatial learning and memory during the developmental stage (Mlsna and Koh, 2013). These behavioral deficits are temporally correlated with the presence of neuronal injury in the following regions involved in modulation of the hypothalamic-pituitary-adrenal stress response (Mlsna and Koh, 2013): (1) the lateral septal nuclei which is involved in motivational and affective function; (2) the amygdala which is involved in anxiety, threat-induced behavioral arousal and emotion; and (3) the ventral subiculum/CA1 which is involved in spatial learning (Mlsna and Koh, 2013).

The functional deficits with ELS may result from short term potentiation (STP) alterations in the PFC. STP of the PFC is important to its functions including short-term working memory, information processing and decision-making processes (Hernan et al., 2013). Using a flurothyl mouse model of ELS, it was observed that recurrent seizures early in development affect STP at Layer II/III (LII/III)-to-LV and LV-to-LV networks in the PFC (Hernan et al., 2013). Both networks are involved in working memory tasks: the apical dendrites of LV neurons serve as the receiver for feedback information from thalamic inputs as well as from cortico-cortical connections in LII/III (Kuroda et al., 1998), and the basal dendrites are integral to the interconnected network of deep pyramidal neurons whose continued firing during the delayed phase of a working memory task is thought to underlie short-term working memory (Hernan et al., 2013). Therefore, alterations in STP in the PFC induced by ELS may lead to cognitive deficits (Deng et al., 2011), particularly in spatial learning and working memory.

JME-related cognitive impairment is associated with cortical abnormalities. fMRI and EEG show gray matter volume alterations in the superior midline frontal regions in JME, with reduced volume seen in some studies (O’Muircheartaigh et al., 2011) and increased volume in others (Koepp et al., 2013). Gray matter of the posterior cingulate and the anterior callosum regions are affected in JME and may contribute to reduction in cognitive performance demonstrated in letter fluency and similarity tasks, concordant with JME frontal lobe dysfunctions (Sonmez et al., 2004). Generally speaking, JME patients have subtle focal cortical abnormalities and gray matter reduction in the mesial frontal cortex, especially the supplementary motor area (SMA) and posterior cingulate cortex. Cognitively, they exhibit impaired verbal fluency, comprehension and expression, as well as impaired nonverbal memory and mental flexibility (O’Muircheartaigh et al., 2011). White matter (WM) abnormalities in these regions and networks are also observed, which we further discuss in the next section.

Besides the cortex, seizure-induced abnormalities in the callosal and cerebellar networks affect cognitive function. In TLE, Schneider et al. (2014) found that anterior and mid-callosal corpus callosum (CC) changes affect cognitive performance. In chronic intractable TLE patients with intermittent explosive disorders, total brain and cerebellar volumes, particularly in the left cerebellum, are influenced by patient age and duration of epilepsy. These alterations in total brain and cerebellar volumes are strongly associated with cognitive impairment, whereas alterations in hippocampal volume have a minor influence on cognitive parameters (Hellwig et al., 2013).

### White Matter Changes

WM is composed of glial cells and myelinated axons and serves as signal transmitter from one cerebral region to another or to lower brain centers. Diffusion tensor imaging (DTI) reveals the following associations between WM in different regions of the brain and cognitive function: (1) right temporal WM with language and executive function; (2) the CC with intelligence and language; and (3) left parietal WM with language (Kim et al., 2012; Widjaja et al., 2013a). WM impairment may reflect connectivity disruptions in cortical processing networks that are necessary for cognitive development (Widjaja et al., 2013a). Here we discuss specific WM changes seen in JME, chronic TLE and CAE.

In patients with JME, WM abnormalities are seen in the bilateral anterior and superior corona radiata, genu and body of CC, cingulum connections to the temporal cortex (Seltzer and Pandya, 2009), posterior parietal regions (part of the splenium) and multiple frontal regions which result in widespread interconnection disturbances in the frontal lobe (Kim et al., 2012). These structural abnormalities in the thalamofrontal network may account for JME patients’ poor performance in frontal functions.

Patients with chronic epilepsy may have cognitive comorbidities and widespread network abnormalities outside the epileptic zone, such as in WM areas that affect cognitive function and global intelligence (Vaessen et al., 2012). In chronic TLE patients, WM abnormalities are seen in the anterior temporal lobe, mesial temporal lobe, ipsilateral cerebellum, posterior regions of the CC, and the frontoparietal lobe contralateral to the side of seizure onset (Riley et al., 2010; Rodríguez-Cruces et al., 2018). Abnormalities in the anterior temporal lobe are correlated with delayed memory, the mesial temporal lobe with immediate memory, and the CC with earlier age of seizure onset (Riley et al., 2010). Although chronic epileptic patients with regional or multi-regional WM impairment usually exhibit severe and complicated cognitive impairment, their whole brain WM volume does not differ from that of healthy controls or of patients with little to no cognitive impairment (Vaessen et al., 2012). This suggests that impaired WM connectivity rather than WM volumetric change is involved in cognitive decline in patients with chronic epilepsy.

WM changes are observed in patients with CAE, a syndrome of idiopathic generalized epilepsy. In untreated CAE patients, there are significant WM abnormalities in the bilateral thalamus, anterior CC, upper brainstem, prefrontal areas, anterior cingulate, parietal areas, posterior cerebellar hemispheres, and in subcortical structures including the bilateral putamen and bilateral posterior limbs of the internal capsule. These changes point to impairment of WM integrity in the basal ganglia-thalamocortical circuit of drug-naïve CAE patients, which may result in increased cortical excitability and cognitive, linguistic and behavioral/emotional deficits during and between seizures (Yang et al., 2012).

## Network and Topological Basis of Seizure-Related Cognitive Outcomes

### Networks

Epileptogenesis engages multiple brain networks. The functionality of these networks is based on the level of synchronous neural activity or structural correlates between different areas through a complex pattern of increased or decreased connectivity (Stam and van Straaten, 2012). The exact number of networks involved and how they interact in epileptogenesis is unclear. Analysis of known networks provides information on: (1) the onset, propagation and termination of seizures (Kramer et al., 2008); (2) the preictal, ictal, interictal and postictal state of functional networks in epilepsy (Horstmann et al., 2010; van Dellen et al., 2012); (3) alterations in structural networks in epilepsy (Bernhardt et al., 2011); and (4) mechanisms of seizure comorbidities such as cognitive decline and behavioral deficits (Vlooswijk et al., 2011; Vaessen et al., 2012). For a detailed discussion on the functional and structural networks involved and topological changes in epilepsy, we recommend reviews by Halász (2010a,b), van Diessen et al. (2013) and Wang et al. (2015). Here we discuss some of the networks associated with seizure-related cognitive dysfunction focusing on the DMN, adaptive networks and the thalamo-frontocortical network.

#### The Default Mode Network: A Resting-State Network

The DMN consists of brain regions that are activated during the resting state and during internally directed cognition (Karmonik et al., 2010) and deactivated during task engagement (Danielson et al., 2011). It has primary nodes (sites or brain areas) in the precuneus/posterior cingulate and the medial frontal and lateral parietal cortices involved in introspective and social cognitive functions (Danielson et al., 2011). The DMN is abnormal in several types of epilepsy, which can be either the cause or the result of seizure-related cognitive comorbidities.

In children with refractory epilepsy, decreased DMN connectivity is seen in the posterior cingulate cortex/precuneus, bilateral lateral parietal cortex, and the anterior and mid-cingulate cortex (Widjaja et al., 2013b). In patients with medial temporal lobe epilepsy (mTLE) and HS, regional synchronization is significantly increased in the ipsilateral parahippocampal gyrus, midbrain, insula, CC, bilateral sensorimotor cortex and frontoparietal subcortical structures, but is decreased in the cerebellum and the DMN, specifically in the precuneus and posterior cingulate gyrus, bilateral inferior lateral parietal, and mesial PFC (Zeng et al., 2013). In absence patients, functional connectivity is increased between the frontal, parietal and temporal lobes but is decreased in the DMN, which may result in cognitive mental impairment and loss of consciousness during an absence seizure (Luo et al., 2011; Li et al., 2015).

DMN abnormalities are seen during resting interictal periods without interictal epileptiform discharges (Luo et al., 2011). In patients with complex partial seizures (CPS), the average volume of activated brain regions is 98% higher than that of controls, and 81% of activated areas are in cognitive regions of the frontal and temporal lobes, anterior cingulate cortex, precuneus and cuneus, while the remaining 19% are in the precentral gyrus, the superior and medial occipital gyrus, the parahippocampal gyrus, the inferior parietal lobule and the angular gyrus (Karmonik et al., 2010). In CPS patients, large areas of activation occur in the frontal and temporal lobes, as well as the cuneus and precuneus, as opposed to the control group where activation is found mostly in the parietal lobe (Karmonik et al., 2010). These results suggest that switching from goal-directed behavior to the default mode in CPS patients is impaired.

In summary, complex partial, generalized tonic-clonic and absence seizures can decrease the activity of the DMN (Danielson et al., 2011). While disorders in other regions may contribute to epileptogenesis and propagation, dysfunction in the DMN appears to be responsible for widespread functional cognitive impairment (Zeng et al., 2013).

#### Brain Adaptive Networks and Connectivities

There is evidence that the brain undergoes structural and connectivity adaptations in response to seizures to preserve cognitive function. We present some of the evidence in this section, with a focus on language preservation in seizures of the left posterior superior temporal gyrus (LSTGp), striatal changes in benign epilepsy with centro-temporal spikes (BECTS) and response to hypometabolism in temporal and frontal lobe seizures.

When the LSTGp is temporarily impaired by a seizure, brain trauma or stroke, the brain adapts to maintain language comprehension ability. Data from repetitive transcranial magnetic stimulation (rTMS) with fMRI studies suggest that this adaptation consists of two parts: (1) increased synchronization between compensating regions coupled with decreased synchronization within the primary language network; and (2) decreased activation at the rTMS site as well as in distal regions followed by a recovery process. Adaptation involves three synchronization centers: the contralateral homolog of the area receiving rTMS (i. e. the right STGp), areas adjacent to the rTMS site and the medial frontal gyrus, a region involved in discourse monitoring (Mason et al., 2014). Because of such insights on the role of the language network in epilepsy-related cognitive outcomes, particularly its adaptive response to injury, this network may be a therapeutic target to prevent cognitive impairment in epilepsy.

BECTS is a benign epilepsy syndrome involving the Rolandic area. It is the most common childhood epilepsy syndrome and is considered a neurodevelopmental disorder with an underlying genetic and anatomical basis (Lin et al., 2012). Children with BECTS show aberrant volume and morphology in subcortical regions involved in motor processing and executive functions with reduced functional connectivity in the Rolandic region. A meta-analysis concluded that BECTS has poor cognition outcomes for language ability, which involves visual processing, auditory processing, single-word reading, expressive and receptive language, verbal fluency, processing speed, fluid reasoning and verbal knowledge (Wickens et al., 2017). Structural changes include putamen hypertrophy, dorsoventral elongation of the left caudate and bilateral putamen, and subnuclei expansion in the ventral and dorsal striatum (Lin et al., 2012). But these alterations may not cause cognitive deterioration but instead be cognitively adaptive, as larger putamen volumes have been linked to better cognitive performance (Lin et al., 2012).

FDG-PET show that 60%–95% of unilateral mTLE patients have significant hypometabolism in the epileptic temporal regions during the interictal period (Trotta et al., 2011). Significant metabolic changes have concomitantly been observed in the surrounding and remote brain regions, including the lateral temporal areas, PFC, frontal lobe, thalamus and even some areas within the DMN. These structures are involved in epilepsy-induced reorganization of neuronal networks and effective connectivity within the mesiotemporal regions or other distant brain areas (Zhang et al., 2010), the latter accounting for some of the cognitive impairment observed in mTLE (Takaya et al., 2009). Meanwhile, functional reorganization/plasticity in the non-epileptic temporal lobe may represent a compensatory mechanism sustaining key cognitive functions such as memory or speech (Bettus et al., 2009; Trotta et al., 2011).

Unlike mTLE, there is no consistent pattern of cognitive impairment seen in FLE patients, although some evidence support the notion that cognitive function is impaired (O’Muircheartaigh and Richardson, 2012). Given that the frontal lobes consist a large proportion of the cerebral cortex and contains rich connections with other brain regions, it is not surprising to either find or not find structure-related cognitive deficits in FLE (O’Muircheartaigh and Richardson, 2012).

#### Thalamo-Frontocortical Network

Thalamo-frontocortical network disorders are seen in JME, which is the most common idiopathic epilepsy syndrome and is considered a benign seizure disorder (Wandschneider et al., 2012). The JME phenotype includes a frontal lobe type neuropsychological profile, photosensitivity, hyperexcitable motor cortex, and abnormal functional connectivity of the motor cortex and SMA (Vollmar et al., 2012). Advanced imaging studies have identified functional and structural abnormalities in the frontal cortex and thalamus in JME patients (Wandschneider et al., 2012). Connectivity is reduced between prefrontal and frontopolar regions and increased between the occipital cortex and the SMA (Vollmar et al., 2012). This may form the anatomical basis of cognitive triggering of motor seizures in JME, as well as the link between seizure semiology, neurophysiology, neuropsychology and imaging findings (Vollmar et al., 2012). A subnetwork comprising the primary motor cortex, precuneus, cerebellum lobules IV and V, basal ganglia, bilateral parietal/postcentral gyrus, subcortical regions and right hippocampus is hyperconnected in JME patients, which is correlated with decreased auditory memory, verbal fluency and executive function (Caeyenberghs et al., 2015). Of note, the highly heritable nature of JME, along with the cognitive dysfunction observed in otherwise healthy siblings of JME patients, supports the concept of a genetically determined thalamo-frontocortical network dysfunction (Wandschneider et al., 2012).

### Topology

Modern brain mapping techniques such as fMRI and DTI suggest that brain function depends on large-scale networks rather than isolated brain areas. Disorders in components of these networks, even a small change in neuronal network topology, can break the network balance, induce explosive synchronization transition and activity propagation, and lead to epileptic seizures (Wang et al., 2017). A topological view of brain functioning borrows concepts from its mathematical counterpart, concepts such as strength, path length, clustering coefficient and efficiency, global efficiency, local efficiency, modularity, hub distribution and small-world. Study methodologies such as region of interest approach, unbiased whole brain approach and graph theoretical analysis are used to study the correlations between network metrics and clinical characteristics. Within this framework, brain images are treated as a close topological space consisting of functional and structural networks or sub-networks associated with cognitive and behavioral functions. Seizures, even psychogenic non-epileptic seizures associated with attention, emotion and sensorimotor systems (Ding et al., 2014), induce an elastic-like deformation in the brain, resulting in alterations of the connectedness, continuity and boundary of areas and networks. In this section, we briefly discuss topological changes in the brain on the network level and the neuronal level.

Recent studies examined topological changes in functional and structural networks and sub-networks in epilepsy and their relation to seizures and seizure-related cognitive outcomes. Bonilha et al. (2014) found that children with new-onset epilepsy have a suboptimal topological structural organization with enhanced network segregation and reduced global integration. This results in: (1) structural reorganization that involves the redistribution of nodes from the posterior to the anterior head regions; and (2) lower IQ and poorer executive function in these children (Bonilha et al., 2014).

The most common epilepsy syndrome in children, CAE, is shown to have altered whole-brain structural network topology, impaired subnetworks in subcortical and orbitofrontal structures, microstructural changes in the genu of the CC and bilateral precuneus. The bilateral precuneus is a core area in the DMN and involved in processing of visuospatial imagery, episodic memory retrieval and consciousness (Liang et al., 2016; Qiu et al., 2017).

Others have found topological alterations in cognition-related brain domains in TLE. Using graph analysis, Kellermann et al. (2016) divided functional brain domains into several modules. They found that TLE patients have poorly segregated cognitive modules compared to non-TLE controls. In the control group, there are numerous segregated, small modules related to cognitive functioning that include: (1) verbal memory; (2) language, perception and intelligence; (3) speed and fluency; and (4) visual memory and executive function, whereas in TLE patients there are fewer, larger and more mixed modules including: (1) verbal memory, visual memory and executive function; (2) speed and fluency; and (3) speed, executive function, perception, language, intelligence and nonverbal memory (Kellermann et al., 2016). This phenomenon may suggest a compensatory mechanism to protect brain networks and neurocognitive function from epileptic impairment, which may be helpful clinically for cognition training and rehabilitation attempts.

Topological changes are also seen in the elements of the network: neurons. Neurons display a wide range of intrinsic firing patterns (van Elburg and van Ooyen, 2010). Burst firing, the generation of clusters of action potentials with short inter-spike intervals, is a relevant pattern for neuronal signaling and synaptic plasticity and is modulated by dendritic morphology and ion channel composition. Computational modeling of neocortical pyramidal cells shows that it is not only the total length of the apical dendrite but also the topological structure of its branching pattern that influence inter- and intra-burst spike intervals and predict whether the cell exhibits burst firing (van Elburg and van Ooyen, 2010). Varying the topology of apical dendritic trees by swapping branches within the tree, all the trees will have the same total length and surface area and differ only in the way their branches are connected. This results in wider connections between pyramidal cells and produce firing patterns ranging from tonic firing to strongly bursting. Only a range of dendritic sizes supports burst firing, and this range is modulated by dendritic topology. Alterations in the size or topology of pyramidal cells, as seen in epilepsy, may change neuronal burst firing patterns and thus affect higher-level information processing and cognition (van Elburg and van Ooyen, 2010).

Because changes in topology may influence seizure spread and seizure-related cognitive impairment, the following questions may serve as future research directions: are there rules or principles that can explain and predict seizure spread and seizure-related cognitive outcomes? To what extent are topological changes in multiple regions involved?

The topographical framework of viewing brain function is still in early development. There is a need for standard analysis methods, as different methods lead to different and sometimes contradictory findings. For example, the definition of network weights, such as the definition of edge weights in DTI network analysis, can change the calculated network properties. Thus more accurate and unified analysis should be a research focus.

## Conclusion

There are still many unknowns in the relationship between epileptogenesis and cognitive and topological alteration. Mechanistic studies thus far have been performed using animal models while observational studies are performed on animals and human patients. Animal models have less heterogenicity than human patients, which is advantageous in mechanistic studies where only one or two factors are examined. There are, however, the following limitations with animal models: (1) human epilepsy includes acquired, idiopathic and cryptogenic causes, whereas animal epilepsy models are mostly induced by chemical or physical methods, which means they cannot completely imitate the pathophysiological course of human epilepsy; (2) chemical or physical ignition affects the whole brain and disrupts the neurogenic niche, which makes it difficult to parse out the contribution of specific regions or structures to cognition; and (3) human beings have higher intelligence and more advanced cognitive functions such as computation, language and integrated analysis, which cannot be studied in animal models, thus animal models are used to study memory, social behavior and recognition. For readers interested in chemosonbulsant-induced epilepsy and cognitive testing in animal models, we recommend a review by Minjarez et al. (2017).

On the other hand, complicating factors in human studies include the following: (1) most epilepsy patients studied are receiving antiepileptic drugs that can result in cognitive impairment. This acts as a confounding factor when we aim to study the direct relationship between epileptogenesis and epilepsy-associated cognitive deficit; and (2) patients who suffer from chronic epilepsy often have comorbid depression and anxiety, which can result in cognitive decline.

Alterations in anatomic structure, networks and topology are associated with seizure-related cognitive impairment. Integrating multiscale imaging approaches such as CT, PET and MRI for macroscopic observation, super-resolution and electron microscopy for microscopic research, and computational tools for analysis, studying structural or sub-structural alterations in the brain is a promising and rewarding approach to understand brain function and dysfunction.

## Author Contributions

QW: conception and design, manuscript writing, final approval of manuscript. CWZ and ZL: conception and design, final approval of manuscript. BX: final approval of manuscript. LF: conception and design, financial support, final approval of manuscript.

## Conflict of Interest Statement

The authors declare that the research was conducted in the absence of any commercial or financial relationships that could be construed as a potential conflict of interest. The reviewer JIA declared a shared affiliation, though no other collaboration, with two of the authors CWZ and LF to the handling Editor.
